# Testicular sperm extraction in men with sertoli cell-only testicular histology - 1680 cases

**DOI:** 10.5935/1518-0557.20190023

**Published:** 2019

**Authors:** Paulo Franco Taitson, Antônio Mourthé Filho, Moacir Rafael Martins Radaelli

**Affiliations:** 1 Human Reproduction Discipline, DCB/ICBS, Pontifícia Universidade Católica de Minas Gerais. Belo Horizonte, MG, Brazil; 2 ICBS, Pontifícia Universidade Católica de Minas Gerais. Belo Horizonte, MG, Brazil.; 3 Urology Department, Medical School, Faculdade Ingá, Maringá, PR, Brazil

**Keywords:** azoospermia, male infertility, testicular failure, sperm retrieval

## Abstract

**Objective:**

To study the outcomes of testicular sperm extraction (TESE) among men with pure Sertoli cell-only histology identified during diagnostic testicular biopsy.

**Methods:**

This retrospective cohort study involved 1680 cases of patients with nonobstructive azoospermia (NOA) diagnosed with pure Sertoli cell-only histology who underwent testicular biopsy with TESE in a reference center in Brazil by a single surgeon. Sperm retrieval rates (SSR) were the main outcome measure.

**Results:**

Overall, 14.83% of patients with Sertoli cell-only had sperm retrieved with TESE in quantity that allowed the performance of ICSI. No differences were observed in SSR based on testis volume (<15 mL *vs.* <15 mL) or serum FSH level.

**Conclusions:**

Patients with Sertoli cell-only histology can be counseled that they have some likelihood of sperm retrieval with TESE. Based on the findings, patients to be submitted to testicular biopsy for histologic analysis may be concomitantly prepared for ICSI with TESE in case sperm is available.

## INTRODUCTION

Cases of azoospermia due to spermatogenic failure, or nonobstructive azoospermia (NOA) affects between 1% to 2% of the male population, and around 15% to 20% of men seeking infertility evaluation (Willott, 1982; Jarow *et al*., 1989). The establishment of NOA is determined by the absence of spermatozoa in the ejaculate despite the integrity of the spermatic pathway. Until recently, this was reported as an irreversible condition (testicular failure), due to congenital and acquired factors (Willott, 1982).

However, with the advent of intracytoplasmic sperm injection (ICSI), the surgical sperm retrieval of testicular spermatozoa has permitted that many men previously considered infertile become parents (Schlegel *et al*., 1997). Although spermatozoa has been observed in biopsies of testes of NOA patients (Jow *et al*., 1993), they are present in isolated foci of spermatogenesis within the testes (Palermo *et al*., 1992; Schlegel *et al.,* 1997). Because of the heterogeneity within the testis of NOA patients, surgical sperm retrieval techniques, including conventional testicular sperm extraction (TESE), testicular sperm aspiration (TESA), and microTESE, have all been successfully used.

Success rates depend not only on the sperm retrieval techniques but also on the histological morphology of the testes. The three most frequent testicular histological patterns are hypoesermatogenesis, cell maturation arrest, and Sertoli cells-only patterns. Currently, testis histology is the only parameter capable of predicting the chance of sperm retrieval in NOA azoospermia (Su *et al*., 1999). Higher sperm retrieval rates (SSR) have been reported in NOA patients presenting hypoesermatogenesis and cell maturation patterns than patients with Sertoli cells-only morphology (Tournaye *et al*., 1997; Kalsi *et al*., 2012; Gul *et al*., 2013; Abdel Raheem *et al*., 2013).

Previous studies have failed to find evidence that could allow a definitive prediction of SSR in men undergoing surgical sperm extraction based on factors such as serum FSH and inhibin-B levels, testicular volume, and testicular histopathology (Ezeh *et al*., 1999; Bohring *et al*., 2002; Boitrelle *et al*., 2011; Ramasamy *et al*., 2013). This reinforces the role of the heterogeneity within the testis, making testicular biopsy neither mandatory or definitive, as it is unlikely to provide prognostic information. Because the testis has a very heterogeneous histology, spermatozoa can be found in different areas of the testicular tissue. Thus, although the likelihood of sperm retrieval in men with Sertoli cell-only pattern and no spermatozoa is lower, paternity remains a real possibility. SRR ranging from 11% to 37% have been previously observed with the use of microTESE (Ramasamy *et al*., 2007).

Sensitivity of microTESE in detecting focal spermatogenesis in NOA patients with pure Sertoli cell-only histology has been shown to be higher than conventional TESE (Colpi *et al*., 2009). Nonetheless, TESE can be performed outpatient, with few resources, at a low cost, low complication rates. MicroTESE, on the other hand requires expensive equipment and expertise that is often not available, particularly in less developed countries.

In this article we report the outcomes of TESE obtained from a large cohort of NOA patients with Sertoli cell-only pattern conducted at a reference center in Brazil, with the intention to provide diagnostic and prognostic information. 

## MATERIAL AND METHODS

### Patient Population

A retrospective review was performed of the charts of NOA patients who underwent TESE by a single surgeon from 1998 through 2018. The study protocol was approved by the Pontifícia Universidade Católica de Minas Gerais Institutional Review Board. Azoospermia was confirmed by analysis of two different centrifuged semen samples according to World Health Organization criteria. Male sample propaedeutics included testicular volume, seminal analysis, FSH and individual clinical history.

### Patient Assessment

Testis volume was measured by use of physical examination with an orchidometer, with the mean volume of both testes analyzed. Testicular histology was determined based on results of testicular biopsies during intraoperative testicular exploration performed with TESE. Patients with strictly Sertoli cell-only histology (no other histologic patterns) on testis biopsy were included in this analysis. Serum FSH levels were obtained without any hormonal medical therapy within 2 months before TESE.

### Surgical Technique

Patients underwent bilateral TESE testicular biopsy for anatomopathological evaluation and study of testicular fragments in the Andrology Laboratory for spermatozoa research. For the TESE technique, open surgery was performed in an outpatient setting with local anesthetic block using xylocaine at 1% without vasoconstrictor. The testis was delivered through a 1 to 2 cm midline scrotal incision, and the tunica vaginalis was opened to directly examine the tunica albuginea and the testis in its entirety. The morphological characteristics of the testicular wraps were evaluated, avoiding traumas and unwanted manipulations. The tunica albuginea was then opened in an equatorial plane under an operative microscope, taking care to avoid injury to the testicular vasculature. After the initial wide incision and exposure of the testis, six fragments (three from each testicle) were obtained. Fragments were very carefully removed to minimize adverse effects, such as hematomas and tissue inflammation (Ramasamy *et al*., 2013).

A random fragment was placed in Bouin's solution and stained with hematoxylin and eosin (Sigma, Brazil) for the anatomopathological study. Approximately 20 seminiferous tubules were analyzed in each section. All stages of spermatogenesis, when present, were counted from stem spermatogonia to spermatozoa (Boitrelle *et al*., 2011).

The remaining four fragments were incubated in culture medium (HTF, Irvine Scientific, USA) to screen the spermatogenic lineage of cells. The fragments were submitted to tubular stretching in a stereomicroscope (D.F. Vasconcelos, Brazil) at 40x magnification, ensuring that the fragment was always immersed in the culture medium. Microscopic analysis (Nikon Microscope, Japan) was performed at 400x magnification by an experienced embryologist for the presence of sperm. If no spermatozoids and/or spermatids were observed, the removal of tissue fragments progressed more inwardly towards the testicular center. At the end of the procedure, the tunica albuginea and the other testicular wraps were sutured with 4-0 needle aspirates. All patients were discharged in the same day of the procedure, receiving only an analgesic and guidelines.

## RESULTS

### Overall Patient Population

A total of 1680 NOA patients, aged 27-52 years, with strictly Sertoli cell-only histology and FSH levels above normal levels were submitted to the TESE technique. Local complications were observed in 49 cases (2.91%), 41 presenting hematoma and 8 scrotal dermatitis. In all cases resolution was spontaneous without impairment to testicular function.

Male propaedeutics demonstrated slightly reduced testes (<15 ml) in most cases (72%) and elevated FSH levels, with a mean of 36.4 mUI/ml. The anatomopathological report indicated a similar histological pattern in most of the fragments studied: seminiferous tubules with varied diameters and heterogeneous parenchyma, coated by Sertoli cells only. The interstitium and Leydig cells showed no changes. Spermatozoa and/or spermatids in quantity that allowed the conduction of ICSI (SSR) were found in 249 patients (14.83%). Table 1 presents the parameters found for positive patients with TESE.

**Table 1:** Parameters found among Sertoli cell-only patients presenting sufficient sperm for ICSI with TESE

Positive patients with TESE		
**No. spermatozoa ** **(mean±SD)**	**No. spermatids** **(mean±SD)**	**FSH** **(mUI/mL)**
10.1±4.2	8.2±4.3	36.4±(12.8)

### Subpopulation Analysis

SRRs were compared for men with normal-volume testes (>15 mL) compared with men with smaller volume testes (<15 mL), with no significant differences between the two groups. Likewise no SSR differences were observed regarding serum FSH level.

## DISCUSSION

Overall, 14.83% of men with Sertoli cell-only pattern identified on testicular histology (TESE) successfully demonstrated spermatozoa and/or spermatids in quantity that allowed ICSI. This result is lower than that reported by recent study (44.5%) using microTESE for sperm retrieval (Berookhim *et al*., 2014).

Azoospermia has been previously divided into two subgroups, absolute and virtual (Tournaye *et al*., 1997). Absolute azoospermia is defined by the absence of sperm throughout the ejaculate, including the pellet resulting from centrifugation. In the case of virtual azoospermia, also described as cryptozoospermia, the ejaculate occasionally presents few spermatozoa, which may not be found when actually required, i.e., on the day of assisted fertilization. For NOA patients, two alternatives to fatherhood can be offered: testicular sperm retrieval, which offer the possibility for the patient to be the biological father; or semen bank, in which case the patient will not be the biological father.

The search for techniques to obtain cells of spermatogenic lineage for *in vitro* fertilization of oocytes with ICSI is not recent. Patients with reports of spermatozoa in the ejaculate may present spermatogenesis in small areas of the testis, which allows the use of these cells in ICSI. NOA diagnosis involves physical examination, two spermograms with a 15-day interval, and hormonal dosages such as serum FSH level. After azoospermia in the ejaculate is confirmed, the patient should be informed on the possibility of undergoing sperm retrieval. Testicular sperm retrieval is an invasive technique, with rare but possible complications for the patient. It is a feasible alternative to patients who wish to be the biological father, and/or are unable to accept semen from a donor.

The most frequent histological diagnosis in NOA patients is Sertoli-cell only. It normally implies in lower SRR when compared to other histological diagnoses. Despite that, because of the heterogeneity of testicular histology, there is a real chance of spermatozoa being found in patients with Sertoli-cells only, as clearly demonstrated in this article (about 15% of patients). The fact that SRR are maintained in men with small-volume testes (Bryson *et al*., 2014) and men with elevated FSH (Ramasamy *et al*., 2009) reflects the fact that these men are still likely to have heterogeneous areas of histology within the testis. This is important information, which could help couples decide on the best option for them.

In the current study SRR did not show any differences based on testis size and serum FSH levels. These results differ from previous studies conducted in men undergoing conventional TESE for NOA, which reported lower SRR in those men with smaller testes (Tournaye *et al*., 1997; Ezeh *et al*., 1999; Bromage *et al*., 2007). However, some recent microTESE studies with a large series of NOA patients demonstrated that testis volume had no effect on SRR (Berookhim *et al*., 2014; Bryson *et al*., 2014). Conflicting results have been reported when FSH was previously investigated as a predictor of sperm retrieval among men undergoing conventional TESE (Chen *et al*., 1996; Jezek *et al*., 1998; Ezeh *et al*., 1999; Boitrelle *et al*., 2011).

The results obtained in the present retrospective study must interpreted with caution. The results are based on the experience of one surgeon, which may differ from other surgeon’s experience. Additionally, although efforts were made to select a random, representative section of the overall testicular parenchyma, the area selected by the surgeon as to the site of testis biopsy may be seen as a potential point of bias. Nonetheless, the large number of patients assessed indicate that men with Sertoli cell-only histology are possible candidates for sperm retrieval using TESE.

## CONCLUSION

The findings obtained from a large cohort of patients presented here can be used to counsel men with NOA. In the presence of NOA and the diagnosis of Sertoli cells-only histology, intra testicular sperm retrieval is a possibility. Therefore, patients to be submitted to testicular biopsy with TESE for histologic analysis may be concomitantly prepared for ICSI in case sperm found.
